# Physical Activity and Cancer Incidence and Mortality: Current Evidence and Biological Mechanisms

**DOI:** 10.3390/cancers17091410

**Published:** 2025-04-23

**Authors:** Joanna Kruk, Basil Hassan Aboul-Enein, Marta Ewelina Gołębiewska, Ewa Duchnik, Urszula Czerniak, Mariola Marchlewicz

**Affiliations:** 1 Faculty of Physical Culture and Health, University of Szczecin, Piastów 40b/6, 71-065 Szczecin, Poland; 2 College of Arts & Sciences Health & Society Program, University of Massachusetts Dartmouth, 285 Old Westport Rd., North Dartmouth, MA 02747, USA; baboulenein@umassd.edu; 3 London School of Hygiene & Tropical Medicine, Faculty of Public Health and Policy, 15-17 Tavistock Place, London WC1H 9SH, UK; 4 Doctoral School of the University of Szczecin, Mickiewicza 18, 70-384 Szczecin, Poland; marta.golebiewska@phd.usz.edu.pl; 5 Department of Aesthetic Dermatology, Pomeranian Medical University, Powstańców Wielkopolskich 72, 70-111 Szczecin, Poland; ewa.duchnik@pum.edu.pl; 6 Chair of Sport Kinesiology, Faculty of Sport Sciences, Poznan University of Physical Education, Królowej Jadwigi 27/39, 61-871 Poznań, Poland; czerniak@awf.poznan.pl; 7 Faculty of Health Science, Department of Skin and Venereal Diseases, Pomeranian Medical University, Siedlecka, 72-010 Police, Poland; mariola.marchlewicz@pum.edu.pl

**Keywords:** physical activity, cancer, incidence, mortality, prevention, mechanisms, review

## Abstract

**Objectives:** There is strong evidence that not enough physical activity is among the most critical risk factors for cancer disease and premature mortality. The literature on the benefits of regular physical activity regarding cancer disease has grown in the last decades. This review aimed to present the current findings on the effect of prediagnosis physical activity on cancer incidence and mortality published between January 2019 and October 2024; this study summarizes the previous evidence, as well as the literature underlying biological mechanisms operating in the exercise–cancer relationship. The review also highlights gaps in the existing research and identifies future research directions. **Methods:** Medline/PubMed, ScienceDirect, and Google Scholar were searched with the search terms “physical activity” and “physical exercise” in conjunction with the MeSH terms for “cancer” and “carcinoma”. Primary, review, and meta-analysis studies published in English were included if they reported a measure of the effect size of prediagnosis physical activity on cancer incidence and/or cancer mortality. **Results:** Evidence from 37 observational studies and 10 reviews were included in this systematic review; 22 studies reported the effect of physical activity on cancer incidence, and 15 studies on cancer mortality. Of the 37 included observational studies, 19 confirmed the previous evidence that physical activity significantly decreased all-cancer-combined and cancer-specific site incidences, and 10 studies focused on cancer mortality. However, the molecular mechanisms involved in this process require future studies. The most convincing evidence maintains the effects of physical activity on body weight and fat, insulin resistance, sex hormones, regulation of redox homeostasis, enhancing the antioxidant defense system, and reducing oxidative stress. **Conclusions:** These data demonstrate substantial prevention against several cancer incidences and mortality among patients who performed regular physical activity, of which dose meets at least the WHO’s guidelines. Further prospective cohort studies and long-term RCT studies are warranted to address a safe and personalized activity dose for cancer-site prevention, identify more precisely the biological mechanisms operating in the physical activity–cancer relationship, and promote the benefits of being physically active.

## Simple Summary

Cancer continues to be a major cause of morbidity and mortality rates worldwide. Estimates have shown that 10 million deaths were attributed to cancer globally in 2020. Consistent data indicate that insufficient physical activity is one of the most important risk factors for cancer. The disease constitutes a public health problem associated with high financial costs, despite the known health benefits of regular physical activity. This review summarizes the current state of the literature on the association between prediagnosis physical activity/exercise and cancer incidence and mortality, the biological mechanisms that are hypothesized, identifies research gaps and methodological limitations of the existing evidence, and outlines the future directions of research.

## 1. Introduction

Consistent data indicate that there is strong evidence that doing enough physical activity can reduce the risk of several chronic diseases, including several types of cancer [1,2,3]. Insufficient amounts of physical activity (PA)/physical exercise (PE) and prolonged sedentary behaviors constitute the main problem of the 21st century, increasing the prevalence of morbidity, premature mortality, and high financial costs of healthcare [4,5]. The term PA describes any forms of bodily movement produced by skeletal muscles in household, occupational, recreational, and transportation settings that result in energy expenditure [6,7]. Physical exercise (PE) is a subset of PA and is defined as planned, structural, and repeatedly practiced bodily movement generated by the contraction of skeletal muscles [8]. PE requires more energy than is released at rest. Exercise is practiced to maintain or improve physical fitness.

The 2020 World Health Organization (WHO) provided public health guidelines regarding regular aerobic exercise (i.e., at least 150–300 min of moderate-intensity exercise, 3–<6 METs, or at least 75–150 min of vigorous-intensity exercise, ≥6 METs, or an equivalent combination of moderate and vigorous exercise) [9,10,11]. In addition, the WHO Expert Panel advocates that adults should perform strengthening exercises at moderate or vigorous intensity at least two days weekly and balance training in addition to aerobic muscle-strength activities to maintain or increase muscular strength and endurance to avoid the onset of chronic diseases, including cancer [12]. Sedentary behavior refers to individuals practicing moderate-to-vigorous physical activity (MVPA) that meets the WHO guidelines, but who also spend a lot of time sitting or lying down (≤1.5 MET) [13]. In turn, the term “physical inactive”, used in many studies, describes individuals who perform insufficient amounts of MVPA to meet daily PA guidelines [13,14]. It is worth mentioning that only 2% of individuals’ waking time is spent in sedentary behavior, and 98% of waking time is spent in sedentary behavior and activity of light intensity [13]. Meeting the WHO guidelines requires energy expenditure of approximately 7.5–14.9 METs/wk or more, whereas a dose of MVPA eliminating a negative effect of a large sitting volume is estimated at ≥35.5 MET-h/wk [13]. Globally, 27.5% of adults do not reach the current public health recommendation for PA (31.7% of women and 23.4% of men) [5]. Epidemiological research has indicated that sedentary behavior is associated with the incidence and mortality of non-communicable diseases, including cancer disease, among other modifiable lifestyle risk factors [9]. There is an estimation that 7.2% of all-cause disease deaths worldwide are attributed to sedentary behavior [15,16]. Cancer is the second leading cause of death worldwide, accounting for an estimated 10.0 million cancer deaths and 19.3 million new cancer cases in 2020 [16]. The respective incidences for selected cancer sites are female breast 11.7%, lung 11.4%, colorectal 10.0%, prostate 7.3%, stomach 5.6%, liver 4.7%, and cancer-related deaths, respectively: lung 18%, colorectal 9.4%, liver 8.3%, stomach 7.7%, breast cancer 6.9%, colon 5.8%. The global burden of cancer diseases has risen and is predicted to reach 28.4 million in the US by 2040 due to the aging of the population. It is forecasted that 73% of these will be ≥65 years [17]. According to the GLOBOCAN 2018 estimates, overall cancer incidence was 2 to 3 times higher in developed countries than in developing countries [16]. The growing literature in this field provides strong evidence for cancer prevention through lifestyle change [18,19,20,21]. The scientific evidence regarding the role of PA in cancer diseases has shown growing progress since 2018, when Roundtable was organized by the American College of Sports Medicine (ACSM) [6,7]. The ACSM delivered guidelines for exercise testing and exercise prescriptions in healthy and cancer cases. There is convincing evidence that PA of moderate-to-vigorous intensity decreases the risk of colon/colorectal, postmenopausal breast cancer, and endometrial cancer incidence and mortality due to cancer [6] and improves the survival of cancer cases [3,22,23,24]. Consequently, adopting the healthiest lifestyle resulted in 17% to 67% lower incident and mortality risks of certain types of cancers [24]. Strong evidence shows that approximately 30–50% of cancers are preventable by a healthy lifestyle, especially by the most fundamental lifestyle factors—PA and immunization [24,25]. There is also a growing number of studies on the role of PA/physical exercise (PE) in cancer treatment to reduce cancer recurrence and mortality, decrease fatigue, and improve the quality of a patient’s life [26,27]. However, whether prediagnostic PA/PE can decrease cancer incidence and add survival benefits for cancer cases remains not established for several cancer sites, as well as the mechanisms operating between PA and cancer disease.

This review aims to present the current state of knowledge regarding the association between prediagnostic PA and risk for cancer incidence and mortality. Thus, this article provides new scientific evidence based on epidemiological research studies published between January 2019 and October 2024 and summarizes previous findings and levels of the scientific evidence evaluated by WCRF/AICR and PAGAC in 2018. This review also focuses on molecular mechanisms linking PA to cancer prevention, underlining the role of oxidative stress (OS) in the disease etiology, highlighting gaps in the existing research, and identifying future research directions.

## 2. Materials and Methods

### 2.1. Search Strategy

Medline/PubMed, ScienceDirect, and Google Scholar (first 280 search results) were searched for relevant publications. For the search, we screened the relevant articles published between January 2019 and October 2024 to check if new findings would change the conclusions of the previous studies published before 2019. The database search was finalized on 31 October 2024. The search was carried out using the Medical Subject Headings (MeSH) terms for “cancer” in conjunction with the MeSH terms for “physical activity”. For elucidation of the cancer–PA association, the following keywords were applied: “total cancer” OR “site-specific cancer” AND “physical activity” OR “physical exercise” OR “sports”. Initial screening of publications evaluated the title and abstract. A secondary screening assessed the articles for relevancy to remove duplicates.

### 2.2. Selection Criteria

All authors reviewed the titles and abstracts to select relevant articles. Due to the large body of articles testing the relation between PA and cancer disease, only studies that met the following established criteria were included in the present review: they (1) were reviews, meta-analyses, and original human studies; (2) they examined the association between PA and risks of cancer incidence and risks of cancer death; (3) they provided objective PA measure or used a valid questionnaire, relative risk or odds risk or hazard ratio (RR/OR/HR) and 95% confidence intervals (CIs) or quantification of exercise impact and were fully adjusted for confounders or matched, and evaluated effect sizes and statistical significance; (4) participants were 18 years of age or older and had confirmed cancer diagnosis; (5) studies had a sample size of ≥ 100 cancer cases (cancer incidence). The review was limited to articles in English. Recently published observational studies and reviews focusing on the biological mechanisms through which PA can exert prevention against cancer were also identified. We also manually reviewed the list of references in the original articles or review articles concerning the relevant topic to broaden the search for previous data.

### 2.3. Data Extraction

Titles, abstracts, and full-text articles were independently evaluated for eligibility and the relevant information for each included study. In case of disagreement during the selection process, the authors discussed the eligibility of a study. For Table 1, data were extracted from systematic reviews, meta-analyses and original studies, PA guidelines, and scientific reports. For updated research, we screened the titles, abstracts, and full text of the identified observational research published between 2019 and 2024 (Table 2 and Table 3). Title, authors, year of publication, journal title, country, sample of participants, research design, instruments used to measure PA, main findings, and conclusions were extracted.

## 3. Results

### 3.1. Selected Articles

**Figure 1:**
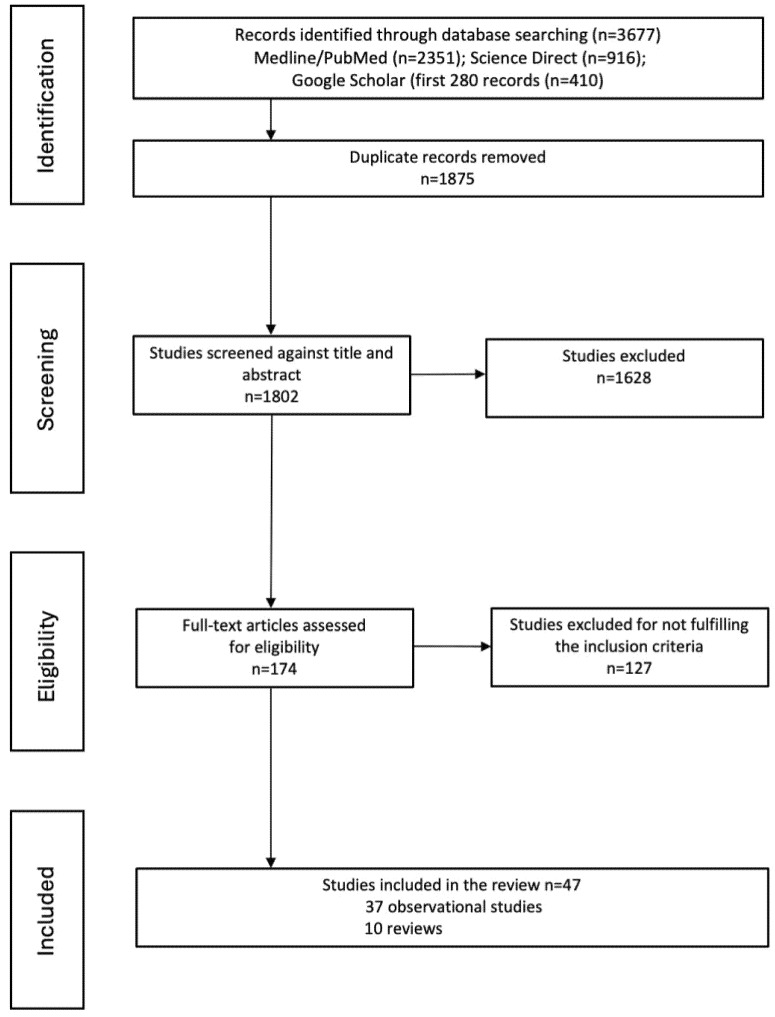
Flow diagram of study identification and selection process.

A total of 3677 articles were selected for this systematic review. After removing duplicates (n = 1875), 1802 articles remained to read titles and abstracts. After eliminating 1628 articles by title/abstract, 174 full-text articles were analyzed. After applying the inclusion and exclusion criteria, a total of 47 articles (39 observational studies and 10 reviews) were included in Table 2 and Table 3. The selection process is shown in Figure 1.

Table 1 lists the research literature findings published before 2019 regarding the effect of PA on cancer incidence and cancer survivors [6,22,23,28]. The percentages of the risk reduction are based on the above-cited articles, which reported statistically significant risk estimates (OR, RR) with 95% confidence intervals for the highest versus lowest level of activity.

Evidence shows that engaging in recommended amounts of PA was associated significantly with a lower risk of 12 different types of cancer incidence by 10–29%; average risk of cancer reduction ranging from approximately 19% to 24% for the colon, 7–13% for breast, 20–27% for endometrium, 15–21% for esophagus, and 10–25% for lung. For liver, kidney, ovary, pancreas, bladder, and gastric cancers, the magnitudes of reductions in risk were as follows: 27%, 12%, 8%, 11%, 15%, and 17%, respectively. According to the World Cancer Research Fund/American Institute for Cancer Research (WCRF/AICR) Third Expert Report 2018 [28], the level of scientific evidence for the association between PA and cancer incidence was categorized (on a five-degree scale) as convincing only for colon cancer and probable for postmenopausal breast and endometrium cancers. The evidence that PA protects against esophagus, lung, and liver cancers was estimated as limited-suggestive. Thus, according to the Panel judgments, there is the strongest evidence for the prevention of colorectal cancer. In turn, the 2018 Physical Activity Guidelines Advisory Committee (PAGAC) Scientific Report [29,30] summarized the scientific literature on the benefits of PA in disease prevention and health promotion. The PAGAC Report indicated that the protection against the incidence of colon/colorectal, breast, endometrium, and esophagus is strong and moderate for lung cancer, whereas only moderate for colon/colorectal, postmenopausal breast cancer, and prostate cancer. Dose–response relationships, mode of PE and its determinants, age, gender, weight, race/ethnicity, and methods applied in PA determination were addressed in these analyses. Also, the PAGAC examined the role of sedentary behaviors in cancer etiology and genetic predisposition.

Findings listed in Table 1 also show that PA is even more effective in preventing risk in cancer survivors, especially for the postdiagnosis activity. Evidence showed the risk reduction in cancer survivors engaged in PA/PE during the cancer prediagnosis period ranging from 10% to 26% for several cancers (colon, breast, esophagus, lung, liver, kidney, prostate, bladder, stomach) when compared to participants who undertook the highest level of assessed PA compared with the lowest level of activity [6,31]. For patients who exercised after cancer diagnosis (colon, breast, esophagus, kidney, prostate), the risk re-duction was observed in 30–69% [6,23,31]. According to the PAGAC Report [29,30], the levels of scientific evidence for the association between PA and cancer mortality were estimated as moderate for colon, breast, and prostate cancer. The PAGAC report included in the analyses self-reported PA, which potentially may suffer from measure error (recall error) and reporting bias [23].

The main characteristics of the current studies included in this review on the effeof PA/PE on cancer incidence and mortality, which met the adopted inclusion criteria, are reported in Table 2 and Table 3. Twenty-two studies addressed the effect of PA on cancer incidence, and fifteen studies addressed the impact of activity on cancer mortality.

### 3.2. Cancer Incidence

Table 2 shows the characterization and results of the association between activity and cancer incidence (n = 22). Regarding the country origin, five studies were conducted in the United States (22.7%), five in China (22.7%), three in South Korea (13.6%), and two in Japan (9%). Of the remaining studies, one article was included in the review from each of the following countries: the UK, Israel, Brazil, Morocco, Nigeria, Canada, Germany, and Spain. Regarding study design, eleven (50%) studies were prospective cohorts, five (22.7%) were case-control, three retrospective cohorts (13.6%), and three studies were cross-sectional (13.6%). The studies examined different types of cancer: breast cancer [33,34,35], colorectal [36,37,38], lung and colorectal [48], lung [43], bladder [32], pancreatic [45,46], ovarian [44], endometrial [39], gastric system [40], and hepatocellular [41,42]. Seven research studies [47,48,49,50,51,52,53] reported data for 2–17 cancer sites, for all cancers combined. Sample sizes of cancer cases ranged from 138 to 22,689. Eighteen studies demonstrated results supporting the beneficial effects of moderate-to-vigorous PA on cancer incidence, reporting statistically significant reduced HRs or ORs. In addition, a case-control study by An and Park reported an increase of 64% in colorectal cancer risk among sedentary individuals at least 10 h/day versus less than 10 h/day [36]. Of the 22 studies, only three found results that did not support the protective effect of PA [44,45,47]. The magnitude of the beneficial effect of PA on cancer-specific incidence outcomes ranged from 4% to 94%. Higher prediagnosis total or recreational PA reduced the incidence of several cancers combined and the following cancer sites: breast, colorectal, lung, hepatocellular, gastric, bladder, endometrial. Incidence of all cancers (total) had the most significant number of contributing studies. One prospective study examined daily intermittent lifestyle PA bouts (≤1 min or ≤2 min) assessed using an accelerometer [53]. The authors found a 31% risk reduction (HR = 0.69; 95%CI: 0.55–0.86) for PA-related cancer incidence (13 cancer sites) for duration bouts (≤1 min) of 4.5 min/day and a 28% risk reduction for bouts 3.7 min/day as a minimal protection, independently on levels of self-reported PA. Objective measures of PA were performed in three studies [38,43,53]. One study [43] (the Mendelian randomization analysis of twelve single-nucleotide polymorphisms linked with PA) examined the causal correlation between activity level and lung cancer, reporting the risk reduction of overall lung cancer by 87%.

Only three studies [39,44,45] provided a separate estimate of the association between moderate-to-vigorous PA during different periods of life and cancer risk. Saint-Maurice et al. [39] examined the effect of five long-term activity patterns between adolescence and the cohort study entry and endometrial cancer risk. The authors found a decreased cancer risk among individuals who maintained high activity levels in adulthood and who practiced activity later in adulthood. Sandhu et al. [45] reported a lack of associations between recreational PA and the incidence of prostate cancer for engaging in activity in the 20 s and 30 s years, mid-adulthood (40 s and 50 s years), and 2 years before cancer diagnosis, independent of the activity intensity. Wang et al. [44] reported no association for moderate-to-vigorous PA at ages 12–13, 14–17, and 18–22 years with the risk of ovarian cancer. Two studies examined the effect of diurnal timing of peak PA on cancer risk reduction [38,51]. Bay et al. [51], using an accelerometer to measure the impact of circadian PA on the risk of cancer, demonstrated the reduced risks of seven out of seventeen site-specific cancer incidences for practicing activity in the morning and afternoon. Also, Stein et al. [38] reported that early-day plus late-day activity was more protective against the risk of colorectal cancer incidence compared to continuous day-long activity. In contrast, the mid-day plus night-time activity did not correlate with colorectal cancer incidence.

In addition, a retrospective cohort study by Chen et al. [41] of 683,157 thyroid cancer cases, not shown in Table 2, who participated in ≥150 min/wk in aerobic activity reported a weak correlation between thyroid cancer incidence and activity level (r = −0.29, *p* = 0.037) but a strong correlation between these variables for the rising trend of this type of cancer incidence (r = −0.65, *p* = 0.04).

### 3.3. Cancer Mortality

Table 3 demonstrates the characterization and results of the prediagnosis PA/PE effect on cancer mortality risk studies.

In the countries where the studies were conducted, almost 53% (n = 8) were performed in the United States, and two of the studies were carried out in the UK. The remaining five studies were from Germany, Australia, China, Colombia, and Canada. Of the fifteen studies, nine (60%) were prospective, and three (20%) were retrospective. The studies examined mortality due to the various types of cancer: breast [54,55], ovarian [58,60,61], endometrial [56,57], lung [59], lung and colorectal [48], and several cancers combined [62,63,64,65,66,67]. The follow-up time ranged from 1 year to 17.6 years.

Samples of deaths due to cancer ranged from 107 to 32,366 individuals. Ten [48,55,56,57,62,63,64,65,66] of the fifteen studies demonstrated statistically significant reduced HRs for cancer-specific mortality for the highest vs. lowest levels of regular moderate-to-vigorous PA. Primarily, strong risk reductions (ranging from 32% to 89%) were reported for the following cancers: breast [54,55], endometrium [56,57]. However, a study by Yang et al. [59] observed a decreased risk for localized lung cancer but not for lung cancer. Moreover, a prospective study by Stamatakis et al. [63] examining daily intermittent lifestyle VILPA bouts (≤1 min or ≤2 min) found a 30–40% risk reduction due to cancer death for a combined 13 cancer sites, of which magnitudes were dependent on activity duration and number of doses. Thus, only four studies included in this review did not support PA’s positive effect on mortality in individuals meeting PA guidelines or being more active [58,59,60,67]. The reported magnitude of the beneficial effect of PA on cancer-specific mortality ranged from 3% to 65%. Higher prediagnosis PA reduced total cancer mortality and the following specific types of cancer: breast and endometrial.

## 4. Discussion

In this review, we analyzed and evaluated the effect of prediagnosis PA/PE on cancer incidence and mortality risks based on 37 observational epidemiological research published between 2019 and 2024 and summarized previous findings on this topic. Regarding the currently published findings, we found that 59% of included studies were published during 2022–2024, which shows growing interest in this topic. Thirty percent of studies were conducted in the USA. The evidence was highly diverse regarding cancer site, study design, location, timing, follow-up periods, PA assessment methods used, quantification of intensity levels, and adequate control for confounding. The studies analyzed different variables of PA, which significantly influenced cancer incidence and mortality risks. Of thirty-seven observational studies included in this review, only three relating to cancer incidence [44,45,47] and four relating to cancer mortality [58,59,60,67] did not find supporting evidence that PA can significantly decrease these risks. The studies in Table 2 and Table 3 confirmed the previous studies (Table 1) that had found that prediagnosis PA can reduce the incidence of cancer-specific sites and mortality. They also extend the findings summarized in Table 1 with several recent results similar or higher in the magnitude of risk reduction, for example, the incidence of colon/rectal [37], endometrium [39], lung [43,50,51] of the cancer types evaluated by WCRF/AICR as having convincing or probable levels of scientific evidence as well as cancer sites combined [52,53]. Moreover, the studies provided new findings on the beneficial effect of PA on the thyroid [68] and hepatocellular cancer incidence [41,42]. Luo et al. [41] found a 40% decreased hepatocellular cancer risk with increased energy expenditure of moderate activity and a 34% risk reduction for brisk walking over 1 h/wk. Still, they observed no preventive effect of vigorous activity on this cancer type.

Regarding the effect of PA on cancer mortality, we also observed risk reductions in studies considering recreational activity, transportation activity, and total activity. The WHO PA recommendation of 150 min/wk of moderate-to-vigorous activity corresponds to an energy expenditure of 8.75 MET-h/wk [69]. We noticed that this dose of activity did not significantly prevent death due to advanced lung cancer but was preventive for localized lung cancer [59]. According to Wilson et al.’s [70] conclusion, an activity dose between 8.7 MET-h/wk and 17.5 MET-h/wk is preventive against cancer incidence and mortality. The researchers found that higher activity doses of ≥22.5 MET-h/wk showed lower prevention of cancer mortality than 15.0–22.5 MET-h/wk; however, they were not harmful.

Our findings supporting the benefits of PA regarding cancer incidence and mortality are consistent with those published in the reviews published between 2019 and 2024 regarding a dose of activity and the protection against carcinogenesis [31,69,71,72,73,74,75,76,77,78]. For example, a review of nine prospective cohorts (755,459 participants, 50 620 incident cancers) by Mattews et al. [71] summarized the effect of PA dose and intensity on the cancer incidence risk. Evidence has shown that engagement in regular recreational activity amount that meets PA recommendations (7.5–15 MET-h/wk) significantly reduced 7 of the 15 cancer types: colon—the decrease in risk ranged from 8% to 14%, breast cancer—from 6% to 10%; endometrial—from 10% to 18%, kidney—from 11% to 17%, myeloma—from 14% to 19%, liver—from 18% to 27%, and non-Hodgin lymphoma—from 11% to 18% in women, confirming the findings shown in Table 1. A meta-analysis study by Franco-Garcia et al. [69] of the research published until February 2019 on the effect of PA on cancer incidence and mortality also underlined the important role of activity dose. The researchers found a significantly decreased risk of total cancer (14 subtypes) incidence (RRs = 0.93–0.85) and cancer mortality (RRs = 0.90–0.82) with increasing dose of activity (4.375–17.5 MET-h/wk). In both cases (incidence and mortality), the lowest preventive dose of activity is smaller than that reported by the study of Wilson et al. [70]. However, these authors observed a nonsignificant correlation between PA and bladder, esophageal, prostate, and rectal cancers.

A recent meta-analysis by Ma et al. [76] found that increased PA significantly decreased the risk of gastric cancer incidence and mortality, RR = 0.8 (0.77–0.90) and RR = 0.76 (0.66–0.89), respectively. The negative correlation exhibited a dose–response association for different PA types and frequency participation.

A quantification of the dose–response relationship between total PA and the risks of cancer incidence (lung, breast, colon, gastric, liver) was also undertaken by a meta-analysis study by Diao et al. [77], based on 98 studies published from January 1980 to 20 March 2023. The highest benefit in risk reduction (14.7%) was demonstrated for lung cancer at 13,200 MET-min/wk of activity. The researchers observed that total PA of the highest levels (≥8000 MET-min/wk) was significantly associated with a reduction in colon, gastric, breast, and liver cancers ranging from 5.1% to 17.1% versus the inactive population. In turn, a quantification of the dose–response of PA–lung cancer association performed by Qie et al. [78], based on 42 cohort studies published up to 17 November 2021, showed a reduction in lung cancer risk by 22% for total PA and a 12% reduction for leisure-time activity. The dose–response curve for lung cancer was U-shaped with a significant benefit-risk reduction at 15 MET-h/wk for leisure-time activity. An earlier systematic review and meta-analysis of 136 studies published before 1 November 2018, by Friedenreich et al. [31], confirmed the improvement of cancer survivors’ health outcomes by prediagnosis and postdiagnosis total and recreational PA for all cancers (11 specific sites) combined and for specific cancer sites. The authors found that recreational PA was more effective in the risk reduction of mortality (HR decreased by 37%) than total activity (HR decreased by 18%). Moreover, the risk reductions observed during postdiagnosis PA for breast and colorectal cancers were more significant than those for prediagnosis PA. The beneficial effect of PA on cancer mortality was observed for PA doses up to 10–15 MET-h/wk by several researchers [9,29,30] supporting the WHO guidelines for cancer survivors’ activity.

Another meta-analysis of eight randomized controlled trials (RCTs) conducted in Germany (n = 3), Australia (n = 2), Canada, the US, and Switzerland (n = 1), published before May 2019 by Morishita et al. [72], reported significantly reduced risk of mortality in cancer cases (breast, lung, hematological malignancy, and others) by 24% (RR = 0.76, 95% CI: 0.40–0.93) as well as recurrence in cancer survivors by 48% (RR = 0.52, 95% CI: 0.29–0.92).

In turn, the current meta-analysis by Takemura et al. [73] of 11 studies (7 nonrandomized trials, 4 RCTs), published on February 3, 2021, on the effects of postdiagnosis PA on the risk of earlier mortality in patients with advanced cancer (lung, colorectal, breast cancer, multiple cancer sites) found that higher level of activity was not significantly associated with a lower risk of death compared to controls. However, when the trials were separated by type, the authors found that a higher level of activity was significantly associated with a lower risk of earlier mortality (Log transformed hazard ratio, InHR = −0.25, 95% CI: −0.44, −0.06) only in nonrandomized trials. The authors have suggested that the observed discrepancies in results from RCTs and nonrandomized trials might result from about twice shorter follow-up time in RCT studies compared to nonrandomized trials (12–35 months vs. 8–74 months, respectively) and potential bias confounding characteristic for observational studies as well as survivals’ disease state.

Another review by Lee et al. [74] (23 studies analyzed), published up to August 2020, reported an increased risk of colon cancer and rectal cancer by 21% and 8%, respectively, among sedentary individuals during work vs. physically active at work. However, additional adjustments for leisure-time activity made the risk estimates nonsignificant.

A current meta-analysis by Yuan et al. [75], based on 14 studies published up to 28 February 2023, also examined total sedentary behavior in endometrial cancer. Evidence showed significantly increased risks of cancer by 22–37% for an occupational domain, 34% for leisure-time activity, and 55% for the highest vs. low level of total sedentary behavior. These findings demonstrate an essential effect of activity dose, follow-up time, and gender, among others.

Our results support the preventive action of PA/PE in developing cancer and death due to the disease, as reported in the reviews mentioned above. However, we found conflicting research results presented in our review compared to those in the reviews [69,73]. In contrast to the review by Franco-Garcia et al. [69], we observed a significantly decreased risk of bladder, prostate, and colorectal cancers. Also, we found conflicting research results between our findings and a meta-analysis study of RCTs conducted by Takemura et al. [73] for lung cancer and multiple-cancer-site prevention. However, there was an agreement in results when the authors examined nonrandomized trials.

The evidence of our review did not show the separate effects of aerobic activity and muscle strengthening (resistance training, weightlifting, pull-ups) on the risk of cancer incidence and mortality. To our knowledge, only a few studies examined these associations. For example, Mazzilli et al. [79] studied weight training about the 10 most common cancer types. The authors observed that weightlifting was associated with a statistically significant decreased risk for colon cancer incidence in individuals practicing low and high weight lifting compared to those who did not lift weights (HR = 0.75, 95% CI: 0.66–0.87, HR = 0.78, 95% CI: 0.61–0.98, respectively). The authors adjusted cancer risks for moderate-to-vigorous PA and other covariates and observed the differences in risk reduction between men and women. Adjusting for aerobic activity resulted in a nonsignificant decrease in low and high weightlifting prostate cancer risks. In turn, Stamatakis et al. [80], using self-reported questionnaires, found that engaging in any strength-promoting exercises (gym-based on own body weight strength activities) was associated with a 31% (HR = 0.69 95% CI: 0.56–0.86) decreased risk of cancer death; practicing the resistance training with the frequency recommended by PA guidelines (≥2 section/wk) was associated with a 34% reduction (HR = 0.66, 95% CI: 0.48–0.92), after adjusting for potential confounders including the aerobic PA, whereas combined association of resistance and aerobic exercises meeting both PA guidelines reduced risk of cancer mortality by 30% (HR = 0.70, 95% CI: 0.50–0.98). Also, Watts et al. [64] reported that playing racquet sports and running were more protective against cancer cases death than aerobic exercise. Chang et al. [65] found a higher risk of death reduction for strenuous sports compared with walking activity. These findings support the benefits of promoting resistance training to prevent cancer development and death.

Joe and Park [81] examined the effect of high-intensity aerobic exercise (90% of maximal heart rate) on cancer suppression in a mouse cancer model. They noticed an approximately 20% decrease in cancer cell viability and an effective decrease in cancer cell profiliation. The authors observed changes in gene expression in muscle, lung, and heart, and the tumor-suppressive effect of high-intensity aerobic exercise depended on PE type, frequency, and type of cancer. In turn, Stteinboim et al. [82] showed that practicing regular high-intensity aerobic PE can reduce the risk of metastatic cancer. According to the authors’ suggestions, aerobic exercise increases glucose consumption of internal body organs, and thus may reduce the availability of energy to the tumor. However, strength training is more effective than aerobic PA in promoting muscle gain, strength, and fat gain reduction, thus supervising glucose homeostasis [83]. This type of exercise can increase the mechanistic target of rapamycin (mTOR) within trained skeletal muscles, hence regulating cell growth followed by an increase in myofibrillar protein synthesis and muscle mass. Independently, endurance exercise increases mitochondrial protein synthesis.

Several studies also reported findings on the joint association of active PA with other healthy lifestyle components (dietary quality, sleep) and cancer incidence [84,85] and death [86], presenting their independent and synergistic associations with overall and site-specific cancer risk.

### Molecular Mechanisms Mediated the Relationship Between Physical Activity and Cancer

Based on epidemiological, clinical, and RCT studies, numerous biological pathways have been hypothesized to explain a decrease in cancer disease risk through regular moderate-to-vigorous exercise (summarized in Table 4) [20,87,88,89,90,91,92,93,94,95,96,97,98,99,100,101].

The commonly proposed mechanisms involve help for individuals to stay at a healthy weight and reduction of body fat [90,101]; the metabolic effects: enhancement of insulin sensitivity, lowering plasma insulin concentration, decrease in insulin-like growth factors (e.g., IGF-1), and increasing level of insulin-like growth factor binding proteins (e.g., IGFBP-3) [97]; increase fatty acids oxidation and ATP formation [20]; effect on carbohydrate metabolism; [98,102]; lowering sex hormones synthesis (estrone, oestradiol, testosterone) [101]; improvement of pulmonary functions and immune system functioning [93]; increase in adiponectin levels [102]; decrease in chronic low-grade inflammation (decreased C-reactive protein, CRP, IL-6, and TNF-α) [103]; reduction of oxidative stress, and up-regulating resistance to it [77,91,99,100]; enhancement of expression of the genes responsible for the production of antioxidant enzymes and DNA repair [87,91,92,104]; inhibition of microsomal prostaglandin E2 (PGF2) (related to greater cancer aggressiveness and weakening the immune system) synthesis [90,101]. Recent studies suggest a strong relation between mitochondrial volume and their biochemical activity, PA, and cancer disease [105]. These organelles have an essential role in energy production, metabolic process regulation, apoptosis, and redox homeostasis. Thus, mitochondrial functioning is essential in cancer immunity and progression [106]. Endurance and resistance PE can repair and eliminate damaged mitochondria, improve their volume and activity [107], and protect against cancer progression and metastasis through a change of cancer cells’ metabolic profile [82].

In the last three decades, there has been growing interest in the role of intracellular reduction and oxidation reactions in inflammatory diseases, such as cancer, as of the processes responsible for the balance between reactive oxygen species (ROS) and reactive nitrogen species (RNS) production and antioxidant defense from enzymatic and non-enzymatic antioxidants [108,109]. The key groups of oxidants are ROS, such as superoxide anion radical (O_2_^●−^), hydroxyl radical (HO^●^), peroxyl radical (ROO^●^), hydrogen peroxide (H_2_O_2_), singlet oxygen (^1^O_2_), hypochlorous acid (HOCl), and RNS like NO^●^ and peroxynitrite (ONOO^−^) [110,111]. These species perform important functions in the organism, essential for maintaining redox balance. Evidence has shown that abnormal endothelial cell functioning is responsible for their dysfunction, resulting mainly from a deficit in the release of NO^●^. This species stimulates mitochondrial biogenesis, controls ROS generation and low-density lipid oxidation, and suppresses inflammation, among others [108,111].

Disruption in the cellular redox homeostasis towards increased activation of pro-oxidant reactions due to the uncontrolled generation of ROS/RNS and other oxidants and/or decreased efficiency of endogenous antioxidant defense systems can lead to OS [108,109,111]. Uncontrolled production of ROS/RNS with the most reactive species HO^●^ results in oxidative damage of DNA, RNA, proteins, carbohydrates, and lipids followed by the initiation of mutagenesis in DNA-repair genes, alternations in the transcription factors (e.g., nuclear factor kappa B, NF-κB; signal transducer and activator of transcription 3, STAT3; hypoxia-inducible factor-1, HIF-1); tumor necrose factor-alpha, TNF-α; nuclear factor—erythroid 2-related factor 2, Nrf2; genetic mutations, and carcinogen metabolizing genes [111,112]. If the disturbed redox equilibrium is persistent or chronic, it generates damaging effects in all cells and tissues. Nucleotide mutation in DNA and oncogenic changes induce a chronic inflammatory microenvironment and the presence of chemokines, cytokines, growth factors, and enhanced production of ROS [112]. Moreover, the induction of cyclooxygenase, COX-2; inducible nitric oxide synthase, iNOS, TNF-α, interleukin (IL) IL-1, IL-6, and expression of mRNAs have been suggested to play an essential role in OS-induced inflammation [109]. TNF-α can induce inflammation and OS on epithelial cells and activate the NF-κB signaling pathway, which is crucial in releasing pro-inflammatory cytokines and chemokines [113,114]. ROS/RNS, as a highly reactive species, triggers the expression of pro-inflammatory cytokines and cell adhesion molecules in endothelial cells and smooth myocytes, remodeling the vascular wall. Inactivation of NO^●^ by ROS, especially O_2_^●−^, and other radicals generated during peroxidation of low-density lipoproteins can change endothelial function. In turn, the uncontrolled reaction of O_2_^●−^ with NO^●^ forms a highly reactive oxidant that is the damaging species ONOO^−^—known as a mediator of protein oxidation and nitration, lipid peroxidation, and mitochondrial dysfunction [111].

A persistent inflammatory environment also involves constant ROS/RNS generation, leading to genomic instability and possible carcinogenesis [111,112,115]. Elevated levels of OS have been well-documented in carcinogenesis [109,112,115]. Evidence has suggested that PA prevents chronic inflammatory diseases and supports its beneficial treatment when applied therapeutically [116]. The effect of PA on OS has been suggested to depend on its mode (e.g., aerobic or anaerobic), intensity (acute or chronic), frequency, duration, muscle contractions (concentric, isometric, eccentric), and subject training [116]. Analysis of increased ROS production by mitochondria related to PA/PE has shown that the increased effectiveness of enzymatic antioxidants buffers this increase, thus destroying the oxidative damaging effect. However, continuous long-term exercise without prior training can move the redox balance toward a pro-oxidative state [77,117,118,119].

Preclinical and clinical exercise research has shown that PE can reduce tumor incidence and tumor growth, inhibit cancer cell proliferation, and induce apoptosis [93,94]. In addition, PE prevents tumor metastasis through several mechanisms, inducing angiogenesis and the activating of tumor suppressors [87,90,95]. Conversely, acute bouts of long-lasting and high-intensity endurance exercise can disrupt redox homeostasis, generating large amounts of ROS/RNS at concentrations exceeding the endogenous antioxidant defense system ability and causing OS in untrained individuals and inducing inflammation through disruption in an expression of leptin, adiponectin, and ghrelin [117]. Evidence has shown that acute long-lasting PE positively correlates with overexpression of IL-1, IL-6, TNF-α, and CRP [118].

Physical activity influences body composition characteristics (e.g., contents of water, fat, and muscles) [119]. It is well recognized that a standardized Phase Angle (PhA) is a good tool for assessing nutritional status and body composition [120]. The parameter indicates cellular health, cellular quality, function, and cell membrane quality [121]. PhA depends on sex, age, and BMI and strongly varies with PA intensity: total PA is associated with larger upper- and whole-body PhA, low-intensity PA—with larger upper-body PhA, moderate-intensity activity—with larger lower and whole-body PhA, whereas vigorous activity does not correlate with PhA [119]. The PhA parameter is easily detected using non-invasive Bioelectrical Impedance Vector Analysis (BIVIVa) [121]. The method allows to measure the electrical integrity of cell membranes, i.e., the preservation of normal cell function and its structure, as well as the ratio of excellular water to total water, including cellular fluid volume balance. Evidence has shown that low PhA value is associated with alteration of fluid balance, including low hydration of cells and their integrity. Thus, muscle-strength performance and the PhA analysis and monitoring of body composition are essential in playing sports [122]. Moreover, low PhA values are associated with OS, the inflammatory markers level, and are a potential marker of inflammation in inflammatory diseases, including cancer [122]. Many studies have revealed that the benefits of regular MVPA are associated with activating the IGF-1/P13/AKT (insulin growth factor 1/phosphoinositide-3 kinase/serine/threonine kinase) pathway [87,89,123,124]. Conversely, long-term pathway activation may be harmful, increasing cancer risk [87]. Studies have shown intense exercise activates the AMPK (AMP-activated protein kinase) signaling pathway, reducing the ATP/AMP ratio [123]. The AMPK pathway activation may suppress tumor growth by the decreased glucose uptake by cancer cells, among others [89,90]. The regulation of MAPK signal transduction and P13/AKT by PA/PE is essential because the signaling pathways promote cancer metabolism, cell growth, proliferation, cell survival, and angiogenesis [123,124,125]. The ability of PE to reduce body fat deserves more attention because adipose tissue acts as an endocrine organ secreting inflammatory hormones (adipokines) and is associated with insulin resistance [126]. These actions include dysregulation of cellular growth, angiogenesis stimulation, and extracellular matrix remodeling, favoring tumor growth and recurrence. Fat cells secrete inflammatory mediators such as TNF-α and IL-6, which promote cancer induction. Insulin resistance elevates circulating insulin and serum glucose levels and enhances inflammation; all these factors can fuel cancer progression [126].

Multifactorial biological actions of PA and accumulating evidence for the primary prevention of cancer incidence and mortality due to cancer, as described above, allowed us to recommend activity as an essential and inexpensive tool to support oncological therapy. A literature review showed that PA that meets or exceeds the public health guidelines for the activity effectively lowers cancer incidence and reduces mortality due to cancer disease. Evidence showed that higher PA amounts in combination with a suitable diet might reduce the side effects of cancer, such as a loss of muscle mass and strength, reduction in mitochondrial biogenesis, anxiety, fatigue, pain, bone density, and sleep quality, among others, and improve tolerance to oncological treatment (chemotherapy, radiotherapy, hormone therapy, immunotherapy), independently on the cancer type and cancer stage [123,127,128,129]. The cancer sides and treatments strongly impact health-related quality of life (HRQoL) and case survival. Regarding the side effects, evidence demonstrates that aerobic activity and resistance training are effective: aerobic exercise decreases anxiety, depressive symptoms, fatigue, and HRQoL and improves physical function; resistance exercise reduces fatigue and improves physical function; combined aerobic and resistance exercises are effective in decreasing anxiety, depressive symptoms, fatigue, HRQoL, and physical function [7]. Thus, supervised, personalized aerobic and resistance exercise programs should become part of the usual care of cancer cases during their adjuvant treatment. The growing literature continues to support exercise interventions in cancer therapy.

Regarding exercise application to clinical therapy, the ACSM International Multidisciplinary Roundtable on Physical Activity on Cancer Prevention on Control (meeting in 2018) supplemented the WHO public health guidelines by prescription aerobic and resistance training specifically for some cancer types, treatments, and health outcomes [7,127]. The guidelines for cancer survivors include aerobic exercise of moderate intensity ≥ three times/wk lasting ≥ 30 min for ≥8–12 weeks and additionally resistance training ≥ two times/wk using ≥ two sets of 8–15 repetitions at least 60% of one repetition maximum [7]. Exercise interventions can improve physical and psychological functioning in cancer cases [127,130].

## 5. Limitations and Recommendations for Future Studies

Like other literature reviews in this field, this review has several limitations. One potential limitation is that the studies included in the review are representative samples of numerous observational epidemiological studies that have reported the association of PA/PE with cancer risk due to our inclusion criteria. Another limitation is that the cancer risk reductions presented in Table 2 and Table 3 originate mainly from observational studies as a source, but not from RCT evidence on the role of PA in preventing/treating cancer. It limits knowledge of mechanisms between PA/PE and inflammation and the power of dose–response relationships.

Another limitation is that the studies included in the PA–cancer association analyses suggest controversial results. Like other authors, we observed more significant risk reductions reported by case-control studies than those reported by cohort studies. This property of case-control studies results from their methodology, selection, and recall bias and is characteristic of this type of research.

In addition, several studies had a retrospective design; thus, a memory bias might accompany this type of study, especially in older individuals. Another influencing factor is the too-small number of studies for most cancer sites, except for colorectal, breast, endometrial, and prostate cancers. Studies imported in this review also suffer from bias because most are due to PA measures using only self-reported survey questionnaires. In addition, most studies did not perform accurate multivariable regression analysis regarding the incomplete assessment of confounding variables, such as lifestyle diet or the control for clinical patients’ characteristics and treatment methods, which can be important potential confounding variables, among others. Physically active individuals are likely to have a healthy diet, lower body weight, and not smoke. Moreover, the studies used different exercise protocols and follow-up periods, exercise types, intensity, duration, daily timing, methods of quantification of activity levels, and several cancer cases; this caused significant heterogeneity among chosen studies and different magnitudes of the presented risk reduction for the same type of cancer.

A significant limitation affecting our findings’ heterogeneity is a cultural/regional response bias. Much research was conducted in diverse cultural countries, using a heterogeneous sample of individuals experiencing cancer. Cancer cases could differ in race/ethnicity, geographic location, socioeconomic status, environmental exposure, national origin, lifestyle, healthcare public policy (e.g., cancer screening program), access to healthcare, and cancer therapy [131]. Only three of thirty-seven studies in our review used the GPAQ to minimize differences in PA assessment before different countries [132]. This review also has strengths because it briefly summarizes the knowledge on the association between PA/PE and cancer incidence and mortality and examines the putative biological mechanisms focusing on the essential role of ^1^O_2_ in inflammation. Another strength of our study is that the assessment of the role of activity in the risk of death due to cancer was based on well-designed prospective studies. We also identified research gaps in this area.

Despite decades of extensive research on the impact of PA on cancer prevention, we identified several significant knowledge gaps in the literature that limit the scientific evidence level for activity benefits. They include study design, the accuracy of PA detection and characterization, i.e., quality (activity type, frequency, intensity, time when activity is measured), and quantification of activity levels. The research used the most subjective measure of activity using self-administered questionnaires, which are less robust in measuring activity of low and moderate intensity and energy expenditure compared to electronic devices (e.g., accelerometers, pedometers). Another gap is no minimum and maximum dose specification and safe activity intensity to achieve cancer prevention, limit cancer progression, and side effects of oncological therapy for specified types of cancer. The next deficiency of epidemiological research studies is a lack of adequate control for confounding variables in the statistical analysis and consideration of effect modification by tumor type and cultural/regional subgroups, e.g., diet, race/ethnicity genetic predisposition, comorbidities, oncological therapy, lifestyle factors, adjustment for other domains of activity.

Another gap is insufficient research data on the importance of proposed biological mechanisms possibly operating in the association between the activity of low, medium, and high intensity and sitting time and cancer development and progression. Several proposed mechanisms listed in Table 4 are interrelated, oppositive, or even may exert synergistic effects. It is important to recognize which mechanisms may be essential in determining the exact type of activity and appropriate dose suitable for reducing specific cancer sites, especially for which the evidence is limited or inaccessible. We also noticed a lack of in vitro model studies for molecular mechanisms regarding the role ^1^O_2_ in the onset and progression of cancer as a potential inflammation enhancer. The gaps mentioned above are like those reported four years ago [101]; this shows that the association between PA and cancer is complex due to the complicated nature of PA variables and the multifactorial nature of cancer.

These findings suggest that in the future, more specific studies on the effect of PA/PE on cancer risk incidence should be continued, especially for less common cancer sites, to ascertain dose–response between PA and sedentary time and cancer associations. Studies should analyze all domains of activity (household, occupational, recreational, transportation) and the main types of exercise (aerobic, strength). In addition, new research should address the personalization of PA/PE dose considering exercise type, individuals’ redox state at rest and adaptation to OS, cancer type and stage of the disease, age, race, ethnicity, socioeconomic status, and obesity, and the influence of other lifestyle-related factors and their changes during life. More well-designed double-blinded high-quality RCTs, animal models, and long-lasting observational studies allow for identifying more specific biochemical and molecular mechanisms operating between PA/PE and cancer disease. To increase understanding of the role of PA/PE in the disease’s etiology and prevention, both self-reported accurate and reliable measures using questionnaires and objective electronic device-based measures of all PA components and levels. Future studies should address the proper quantification of exercise levels.

## 6. Conclusions

This article summarizes the findings of the dispersed scientific literature on the association between PA and cancer morbidity and mortality. Evidence from the previous research literature showed statistically essential reductions in the risk of 12 types of cancer in incidence and mortality due to PA. For colon cancer, scientific evidence of the association between PA and cancer incidence and mortality is the strongest (convincing) and most probable for postmenopausal breast and endometrial cancers. Evidence presented more significant mortality risk reductions among cancer survivors for PA performed before cancer diagnosis. We found that the current evidence generally supports previous risk reductions of morbidity and mortality for several cancer sites among individuals engaging in regular MVPA in an amount that at least meets the PA recommendation given by WHO. However, sporadically, PA/PE did not show any significant protection from cancer morbidity and mortality. This study provides updated evidence that sedentary behavior and obesity are associated with a higher risk of cancer incidence and death. Evidence showed that the effectiveness of PA’s benefit may depend on the type and domain of activity and its intensity, population, lifestyle, activity timing in life, timing of exercise within the day, and type of cancer. However, the findings are limited and inconclusive in the recommendation. We noticed different cut-off points while quantifying PA levels and a considerable heterogeneity among selected study designs and activity measures. Our findings suggest that engaging in MVPA may attenuate the cancer risks of high sedentary time. In addition, we noticed that not only is MVPA important in cancer prevention, but it also provides light-intensity activity, e.g., walking.

Despite the limitations, the present review found that sedentary behavior may be essential in cancer disease incidence and mortality. Compared to the previous systematic reviews, we updated the literature findings, including several new research articles regarding preventive amounts of PA. Evidence for the underlying biological mechanisms involved in the PA–cancer relationship is accumulating, including an important role of ^1^O_2_ in the disrupting of cellular homeostasis and inflammation, thus, in cancer disease. Due to the regulation of ROS/RNS levels and its essential role in intracellular signaling pathways, PA is a critical factor influencing cellular redox homeostasis.

Despite strong evidence of elevated levels of OS and DNA damage in several human cancers, the exact mechanisms linked with exercise that influence redox homeostasis require future studies due to the complexity and multifactorial dependence of both the cancerogenesis process and PA. Regardless of the immense knowledge of health benefits and reported high potency of cancer prevention and treatment by PA/PE, the current knowledge is still insufficient to define recommendations regarding the effective but safe type of PE and its dose for different cancer types. Future studies may support healthcare professionals in increasing individuals’ awareness of the health benefits of regular PA and prescript exercise programs for individual patients as inexpensive means to prevent cancer incidence and progression.

## Figures and Tables

**Table 1:** Summary of epidemiologic evidence on the association between physical activity and cancer incidence and cancer-specific mortality.

Cancer Site	Magnitude Risk Reduction (%)			Level of Scientific Evidence		
	Incidence	Survival		Incidence		Mortality
		Prediagnosis PA	Postdiagnosis PA	WCRF/AICR [28]	PAGAC [29,30]	PAGAC
Colon/colorectal	20 ^a^, 24 ^b^, 19 ^c^	23 ^b^, 20 ^d^	30 ^b^, 38 ^d^	Convincing	Strong ^+•^	Moderate
Breast premenopausal	7 ^a^			Probable ^#^		
Breast postmenopausal	13 ^a^, 13 ^b^	18 ^b^, 14 ^d^	31 ^b^, 37 ^d^	Probable	Strong ^+^	Moderate
Endometrium	27 ^a^, 20 ^b^, 20 ^c^			Probable	Strong ^+•^	
Esophagus	15 ^a^, 21 ^b^, 21 ^c^	23 ^d^	69 ^b^	Limited-suggestive	Strong ^+^	
Lung	10 ^a^, 24 ^b^, 21–25 ^c^	22 ^b^, 19 ^d^		Limited-suggestive	Moderate ^•^	
Liver	27 ^b^	22 ^d^		Limited-suggestive		
Kidney	12 ^b^	50 ^b^, 19 ^d^	43 ^d^	Insufficient		
Ovary	8 ^c^			Insufficient	Limited ^−^	
Prostate	contradicting results	∗ ^b^, 10 ^d^	33 ^b^, 38 ^c^, 30 ^d^	Limited-suggestive	Limited ^−^	Moderate
Pancreas	11 ^c^			Insufficient	Limited ^−^	
Bladder	15 ^b^	23 ^d^		Insufficient	Strong ^+^	
Stomach (gastric)	17 ^b^	26 ^d^		Insufficient	Strong ^+^	
All cancers combined	10–20 ^c^, 29 ^e^	18 ^d^	37 ^d^			

The letters a–e indicate that data are acquired from References [6,22,23,28,31], respectively; # deals with vigorous activity only; ∗ means that PA protection against prostate cancer was elusive; + shows the direct dose–response association; ^•^ signifies positive correlation of sedentary time with higher risk; ^−^ suggests dose–response association.

**Table 2:** Main characteristics of the included observational studies on the effect of prediagnosis physical activity on cancer incidence risk.

Author, Year, Country	Study Design/ Population, Number of Cases/Age/Period	Physical Activity Assessment Methods (Type of Activity, Detection)	Main Results (OR/HR, 95% CI)
bladder			
An et al., 2024, Japan [32]	Prospective 50,374 individuals aged 40–79 years, 153 bladder cancer cases, follow-up 17.5 years	Japan Collaborative Study for Evaluation of Cancer questionnaires; determination of weekly duration of practicing sports or physical exercise, and sitting or reclining time over the past year or two, and occupational activity	Recreational sports participation of 5 h/wk vs. 1–2 h/wk; HR = 0.28 (0.09–0.89), *p* for trend 0.017, occupational PA (standing and walking), HR = 0.53 (0.32–0.85) vs. mostly sitting at the workplace. Protection stronger among men
breast cancer			
Bigman et al., 2022, Nigeria [33]	Case-control; 508 breast cancer cases, 892 controls; mean age 45.5 and 40.1 years; 2014–2016	Leisure-time PA (aerobic and resistance) based on questionnaire. Face-to-face interview, MET-h/wk calculated in the past year and divided by quartiles (Q_1_ < 3.75, Q_2_: 3.75–6.69, Q_3_: 6.70–14.74, Q_4_ ≥ 14.74)	OR = 0.51 (0.27–0.96) for Q_4_ vs. Q_1_; risk reduction varied by cancer subtypes and was more expressed in non-obese women
Fortner et al., 2024, USA [34]	Retrospective Nurses’ Health Studies, 187,278 women, 12,785 breast cancer cases, aged 30–55 and 25–42 years, 1986–2016 and 1989–2017 follow-up, respectively	Self-administered questionnaire, recreational PA (ten categories) reported every 4 years. Energy expenditure estimated by MET-h/wk for MVPA as annual average	ER+/PR+ breast cancer: ≥27 vs. <3 MET-h/wk: premenopausal women, OR = 0.83 (0.70–0.99), postmenopausal women OR = 0.86 (0.78–0.95) (total recreational activity). MVPA: premenopausal women OR = 0.88 (0.69–1.11), postmenopausal women OR = 0.71 (0.58–0.88). No association for ER-/PR—breast cancer
Liu et al., 2024, China [35]	Cross-sectional 233 breast cancer cases, 6395 controls from NHANES aged ≥ 20 years, 2011–2020 follow-up	Global Physical Activity Questionnaire (GPAQ), PA classification: vigorous work activity, moderate work activity, walking/cycling, vigorous leisure activity, moderate leisure activity during 7 last days (MET-min/wk), total activity level classification: low < 600, light 600–<1800, moderate 1800–<3000, high ≥ 3000	Women light active, OR = 0.95 (0.68–1.34); moderate active, OR = 0.92 (0.57–1.49); high active, OR = 0.56 (0.37–0.86), *p* for trend 0.014.
colorectal			
An and Park, 2022, Korea [36]	Cross-sectional; 33,403 participants, 193 colorectal cancer cases, aged ≥ 20 years; 2014–2019	Self-administered GPAQ; recreational, occupational and transportation in three levels: sedentary behavior, moderate intensity, vigorous intensity, according to WHO recommended standard for activity, and sitting/reclining time evaluated. Sitting behavior dichotomized into <10 days and ≥10 days monthly	Individual with long sitting time (≥10 h/day vs. <10 h/day, OR = 1.64 (1.22–2.01)); No significant relation between colorectal cancer and the different domains of PA
Hatime et al., 2022, Morocco [37]	Case-control; 1516 case-control pairs; colorectal cancer; September 2009–February 2017	Self-administered GPAQ; Occupational, household and leisure-time activity (last 7 days) (MET-min/wk) three levels: low intensity < 600, moderate 600–3000, vigorous ≥ 3000	Vigorous PA vs. low intensity, OR = 0.77 (0.62–0.95) colon *p* for trend 0.05, OR = 0.65 (0.53–0.80) rectal *p* for trend 0.001, OR = 0.71 (0.61–0.82) colorectal *p* for trend 0.09 Sitting time ≥ 4 vs. <4 h/day: OR = 1.02 (0.87–1.20) colon OR = 1.17 (0.99–1.37) rectal OR = 1.09 (0.97–1.22) colorectal
Stein et al., 2024, Germany [38]	Prospective, 86,252 participants from UK Biobank aged 42–79 years, 529 colorectal cancer cases, 5.3-year follow-up	Accelerometer (Axivity AX3 wrist—worn triaxal), functional principal component analysis (fPCA) used to ascertain diurnal timing of PA patterns between 2013 and 2015	Continuous day-long activity, HR = 0.94 (0.89–0.99) for higher vs. lower overall; early plus late-day activity vs. mid-day, HR = 0.89 (0.80–0.99), late-day activity vs. early-day, HR = 0.93 (0.85–1.02) mid-day plus night-time activity vs. early/late-day, HR = 1.02 (0.88–1.19)
endometrial			
Saint-Maurice et al., 2021, USA [39]	Prospective cohort; 67,705 women, 1468 endometrial cancer cases, aged 50–71 years; 12.4-year follow-up period	Risk Factor Questionnaire identification of five long-term leisure-time PA patterns between adolescence and the cohort study entry at ages 15–18, 19–29, 30–35, and 10 years before cohort entry. Weekly duration of PA for each age period rarely or never, 0.5–<1 h, 1–3 h, 4–7 h, ≥7 h	High level PA (6–7 h/wk) over time, OR = 0.81 (0.67–0.98); low level PA (1–2 h/wk) over time, OR = 0.85 (0.69–1.04), increased activity level, OR = 0.74 (0.61–0.91); decreased activity level, OR = 0.98 (0.80–1.19) vs. <1 h/wk at each age period
gastric			
Fagundes et al., 2021, Brazil [40]	Case-control; 147 gastric cancer cases, 150 controls; July 2017–April 2018	Baecke Physical Activity Questionnaire; self-reported level of occupational, leisure-time, and transportation activities during three periods of 5, 10, and 15 years before the cancer diagnosis specified in three levels	PE performed 5 years before diagnosis: OR = 0.29 (0.12–0.75) for 1.75–2.00 and leisure and locomotion PE, OR = 1.66 (0.62–4.44) for 2.00–4.75 vs. 1.25–1.75. For 10 years before diagnosis, OR = 0.24 (0.09–0.69) for >3.25–4.50, for 15 years, OR = 0.22 (0.08–0.68) for >3.50–5.00 compared to 1.50–2.75 level
hepatocellular			
Luo et al., 2020, USA [41]	Prospective cohort; two cohorts: the Nurses’ Health Study and Health Professionals Follow-up Study; 122,075 participants: 44,540 men, 77,535 women aged 40–75 years; 138 hepatocellular cancer cases; 23-year follow-up	Biennal questionnaire. Average time per week spent walking, jogging, running, swimming, bicycling, calisthenics and other aerobic exercise, squash/racquetball, tennis, weightlifting, chopping/digging, number of stairs climbed, yoga, stretching, and toning, estimated in MET-h/wk. Total activity coded into three-levels	Total PA, HR = 0.78 (0.51–1.18); moderate intensity activity: HR = 0.60 (0.38–0.94), *p* for trend 0.04 vigorous intensity, HR = 0.88 (0.56–1.37) highest vs. lowest tertile; brisk walking over 1 h/wk vs. non-brisk walking, HR = 0.56 (0.35–0.90) *p* for trend 0.006.
Han et al., 2024, South Korea [42]	Retrospective National Health Insurance Service cohort of 1439,152, 22,689 hepatocellular cancer cases in diabetic patients mean age 58.1 years, 5.2-year follow-up period	PA estimated in 2009 and 2011 using questionnaires. Dose of PA assessment in MET-min/wk: sedentary behavior < 500; moderate active 500–1500; active > 1500. Change in PA levels according to change of activity between 2009 and 2011: persistently sedentary; newly active, active, and persistently active	Moderate active, HR = 0.96 (0.93–0.99), active, HR = 0.95 (0.91–0.99) vs. sedentary group. Persistently active behavior vs. persistently sedentary group, HR = 0.91 (0.84–0.98), dose-dependent effects
lung			
Chen et al., 2024, China [43]	Mendelian randomized 11,348 lung cancer cases, 15,861 controls	Self-report questionnaire and objective measure (accelerometer or wearable activity monitor) of moderate-to-vigorous PA duration (minimum of 30 min) of high-intensity activity. Moderate-intensity PA included brisk strolling, recreational sports, and moderate aerobic exercise. Mendelian randomization	Overall lung cancer, OR = 0.129 (0.021–0.779); lung adenocarcinoma and squamous cell lung cancer, OR = 0.045 (0.003–0.677). Strenuous sports effect, OR = 0.054 (0.010–0.302)
ovarian			
Wang et al., 2021, USA [44]	Prospective cohort; 84,785 participants, two cohorts of Nurses’ Health Study 28,232 and 56,553, median age 69 and 42 years, respectively, 227 ovarian cancer cases; 15.1-year follow-up	Self-reported average weekly duration of transportation, moderate recreational PA (walking, cycling, hiking, yard work) and strenuous recreational activity (running, aerobics, lap swimming) at grades 7–8 (ages 12–13), grades 9–12 (ages 14–17), and ages 18–22. Total PA score weighted by intensity (MET-h/wk)	PA at ages 12–13, 14–17, and 18–22 years: HRs: 1.34 (0.87–2.05), 1.21 (0.77–1.89) and 1.08 (0.65–1.80), respectively, PA across all these periods, HR = 1.24 (0.80–1.92) for ≥78 vs. <24 MET-h/wk
pancreatic			
Sandhu et al., 2020, Canada [45]	Case-control; 315 pancreatic cancer cases, 1254 controls aged 40–60 years; February 2011–January 2015	Self-administered questionnaire applied to examine trajectories of moderate and vigorous recreational and occupational PA during participants’ 20s and 30s, mid-adulthood (40s and 50s), and 2 years ago. Estimated total weekly MET scores for combined moderate and vigorous activity	Life-course PA trajectories: low activity at all ages, OR = 1.11 (0.75–1.66), increasingly active, OR = 1.11 (0.56–2.21), high active in young adulthood and less in older adulthood, OR = 0.98 (0.62–1.53), and persistently high active, OR = 1.50 (0.86–2.62)
Park et al., 2022, Korea [46]	Retrospective cohort; 220,357 participants, 377 pancreatic cancer cases, mean age of 64.8 years; 4.38-year follow-up	Self-reported IPAQ short form assessed weekly frequency and durations of vigorous PA > 20 min (heavy lifting, digging, aerobic, fast bicycling) during the last 7 days; estimated total MET-hours. Four levels frequency of vigorous activity	HR = 0.47 (0.25–0.89), *p* for trend 0.014 for performing vigorous activity 6–7 days/wk vs. those declared no vigorous intensity PA
combined cancers			
Ihira et al., 2019, Japan [47]	Prospective cohort; 76,795 individuals 36,670 men, 40,125 women, aged 45–74 years; cancer cases: 202 kidney, 373 bladder, and 83 upper urinary tract; 15.1-year follow-up	Self-administered PA questionnaire; Average time per day spent engaged in strenuous exercise, heavy physical work or walking and standing, and sitting time, estimated total METs/day score stratified in tertile. Leisure-time exercise, sports also stratified by weekly frequency	HRs for kidney, bladder, and upper urinary tract cancers: total activity 1.05 (0.74–1.49), 1.06 (0.81–1.39), 0.80 (0.49–1.35), leisure-time sports or PE: 0.87 (0.55–1.38), 0.95 (0.69–1.39), 0.81 (0.39–1.70), respectively, for the highest tertile vs. the lowest tertile
Marshal et al., 2019, USA [48]	Retrospective cohort; Henry Ford Exercise Project; 49,143 adults (mean age 54.0 years); 294 lung cancer and 188 colorectal cancer cases; followed ≥2 and ≥5 years, respectively; 46% women, 54% men; 7.7-year follow-up	Bruce protocol treadmill exercise stress test (pick METs) testing from 1991 through 2009 based on achieved speed. Calculated in MET by Quinton Controller and equations according to ACSM’s guidelines for exercise	Lung cancer: HR = 0.28 (0.17–0.46) (followed ≥ 2 years); HR = 0.27 (0.15–0.49) (followed ≥ 5 years) for the highest (≥12) vs. the lowest (<6) MET tertile, *p* for trend 0.01; colorectal cancer: HR = 0.32 (0.17–0.60) (followed ≥ 2 years) and HR = 0.30 (0.13–0.68) (followed ≥ 5 years) for ≥12 MET vs. <6 MET
Pang et al., 2021, China [49]	Prospective cohort; 460,937 participants, 22,012 cancer cases aged 30–79 years, (liver cancer, gallbladder cancer, biliary tract cancer); 10-year follow-up period	Self-administered questionnaire used in European Prospective Investigation into Cancer with additional modification that included occupational, commuting, household and leisure-time PA during the past 12 months; estimated in MET-h/wk	Liver cancer, HR = 0.81 (0.71–0.93); gallbladder cancer, HR = 0.51 (0.32–0.80); biliary tract cancer, HR = 0.53 (0.38–0.78), for the highest vs. the lowest quartile of total activity
Su et al., 2022, China [50]	Prospective study; 52,938, cancer-free individuals aged 30–79 years, 3674 cancer cases (lung, colorectal, liver, breast, esophageal, stomach); 10.1-year follow-up 2004–2008	Self-reported information on occupational, recreational, and household activities collected by interview-administered questionnaire; estimated in quartiles of MET-h/day, sedentary leisure time quantified in h/day	Highest quartile vs. the lowest quartile, HRs: 0.89 (0.81–0.99) (total cancer); 0.75 (0.60–0.94) (lung cancer); 0.74 (0.55–1.00) (colorectal cancer). Lower risk magnitudes for females and never smokers
Bai et al., 2024, China [51]	Prospective 96,687 participants, 5995 several cancer-site cases; mean age 55.9 years, 7.1-year follow-up	Accelerometer measured PA over 7 days. Circadian patterns of activity delineated through PA trajectories for every 24 h acceleration data. Hourly mean acceleration, peaks (denoting intensity activity) and area under the curve (total PA volume), and the trajectory trend were measured	Vigorous activity pattern, HRs: 0.58 (0.04–0.86); bladder—0.58 (0.04–0.86); breast—0.73 (0.60–0.89); kidney—0.45 (0.26–0.78); lung—0.59 (0.41–0.84); myeloma—0.49 (0.27–0.88); oral and pharynx—0.51 (0.26–0.98), and 0.71 (0.54–0.93) for colorectal, in two distinct peaks of PA levels morning and afternoon
Franco-Garcia et al., 2024, Spain [52]	Cross-sectional 17,704 malignant cancer cases (men and women), median age 47 years October 2016, October 2017, follow-up	ENSE Adult Questionnaire PA levels (PAL): Inactive, Walkers, Actives, Very Actives, scores calculated on the basis of number of days/wk, duration and intensity of activity	Physically active group, OR = 0.62 (0.48–0.80); very active, OR = 0.32 (0.22–0.47), vs. sedentary group
Stamatakis et al., 2023, UK [53]	Prospective cohort; UK Biobank Accelerometry Subsample, 22,398 nonexercising adults (45.2% men, 54.8% women), 2356 total incident cancer cases (13 cancer sites) and 1084 individuals owing to PA-related cancer; mean age 62.0 years; 6.7-year follow-up	Daily vigorous intermittent lifestyle PA(VILPA) self-reported ≤ 1 min and ≤2 min duration bouts assessed using accelerometer	Median daily VILPA duration bouts (≤1 min) of 4.5 min/day. HR = 0.80 (0.65–0.92) for total cancer incidence and HR = 0.69 (0.55–0.86) for PA-related cancer. Minimal protection doses: 3.4 min/day for total cancer incidence, HR = 0.83 (0.73–0.93) and 3.7 min/day for PA-related cancer incidence, HR = 0.72 (0.59–0.88)

**Table 3:** Characteristics of observational studies on the effect of prediagnosis physical activity/physical exercise on cancer mortality risk.

Author, Year, Country	Study Design/Population, Number of Cases/Age/Period	Physical Activity Assessment Methods (Type of Activity, Detection)	Main Results
breast cancer			
Jung et al., 2019, Germany [54]	Prospective cohort; 2042 women from two regions with breast cancer; Vital status assessed in 2009 and 2015, 114 deaths from breast cancer; Age 50–74 years; 6-year follow-up	Telephone interviews based on questionnaire. PA index based on walking, commuting/transportation cycling, recreational activities, sports, and fitness from the age of 50 until diagnosis; leisure-time activities estimated in MET-h/wk: nonparticipant—0; low activity—>0–<7.5; sufficient—≥7.5	HR = 0.54 (0.30–1.00) for increasingly active women. For decreasingly active from pre- to postdiagnosis, HR = 0.80 (0.45–1.42), sufficient activity in prediagnosis 0.90 (0.55–1.46)
Cannioto et al., 2023, USA [55]	Prospective cohort; 1340 women, 873 with hormone-receptor positive breast cancer, 222 deaths; mean age 50.89 years; study January 2005–December 2010, 7.7-year follow-up time updated through December 2018	Interview-administered questionnaires meeting PA AICR and ACS guidelines, MVPA quantifications: inactive—no MVPA, insufficient—<7.5 MET-h/wk, meeting PA guidelines—≥7.5 MET-h/wk	For meeting PA guidelines, HR = 0.56 (0.41–0.76), *p* < 0.001 vs. no MVPA, insufficient activity, HR = 0.73 (0.52–1.03), *p* < 0.07 vs. no MVPA practice
endometrial			
Friedenreich et al., 2020, Canada [56]	Prospective cohort, 425 women with endometrial cancer (2002–2006, observed to 2019), 18 deaths; age 30–80 years; 14.5-year follow-up, 60 deaths	Interview-administered LTPAQ. Frequency, duration, and intensity of occupational, household, and recreational PA from childhood until diagnosis estimated as average MET-h/wk/yr. Activity classification (MET): light (<3), moderate (<3–5.9), vigorous (≥6). Sedentary behavior in occupational activity (≤1.5)	Higher recreational activity > 14 vs. ≤8 MET-h/wk/yr, HR = 0.54 (0.30–0.96), *p* for trend 0.04. Recreational PA from pre- to postdiagnosis HR = 0.35 (0.18–0.69),
Gorzelitz et al., 2022, USA [57]	Population-based cancer registry, 745 endometrial cancer survivors, mean age 40–79 years; 1991–1994	Self-reported frequency of MVPA, interview (number of session/wk) at ages 12, 20, and 5 years pre-interview. Specification of PA: vigorous (running, lap swimming, basketball, gymnastics), moderate (volleyball, softball, brisk walking, leisurely biking)	HR = 0.61 (0.41–0.92) for women engaged in one MVPA session per week 5 years before diagnosis vs. nonparticipants. For one session of activity engaged at ages 12 and 20 years, HR = 0.95 (0.86–1.06) and HR = 0.87 (0.65–1.16), respectively
ovarian			
Zamorano et al., 2019, USA [58]	Retrospective cohort; Women enrollment into NIH-AARP Diet and Health Study; 566,398 individuals: 339,666 men and 226,732 women, 489 of 741 cases of epithelial ovarian cancer included in analysis; mean age 62.7 years; One-year follow-up	Self-administered questionnaire; questions on intensity and frequence of light and vigorous PA during the past 10 years, estimated in times per week or month. Vigorous activities ≥ 20 min duration and increase in heart rate or heavy sweating.	Frequency ≥ 5 times/wk, HR = 1.03 (0.76–1.39), *p* for trend 0.74 PA in past 10 years: light intensity for ≥7 h/wk, HR = 0.84 (0.48–1.47), *p* for trend 0.50; vigorous intensity, HR = 0.95 (0.65–1.39) HR = 0.60 (0.41–0.0.87) 4–7 h/wk, *p* for trend 0.06, vs. never/rarely practice
lung			
Yang et al., 2022, USA [59]	Record linkage 11 cohorts (7 US, 2 European, 2 Asian); 1588,378 participants, 20,494 lung cancer cases, 13,596 deaths due to lung cancer; One-year follow-up	Self-administered LTPA valid cohort questionnaire; quantification of regular engagement in exercise and sport activities in MET-h/wk based on PA guidelines: none MET (nonparticipants referent), >0–<8.3 (low active), 8.3–16.0 (moderate active), >16.0 (highly active)	Lung cancer specific energy expenditure: 0–<8.3 MET-h/wk HR = 1.00 (0.96–1.05); ≥8.3 MET-h/wk HR = 0.99 (0.95–1.04); localized lung cancer, HR = 0.84 (0.68–1.04) and HR = 0.80 (0.65–0.99), respectively
ovarian			
Hansen et al., 2020, Australia [60]	Prospective cohort; 18 major Australian treatment centers, 958 women with invasive epithelial ovarian cancer; age 18–79 years; January 2012–May 2015	Active Australia Survey, three specific levels (MET-h/wk): least active (0–≤10.5), second tertile (>10.5–≤29.3), most active (>29.3)	HR, second tertile 0.98 (0.74–1.30), third tertile 0.93 (0.79–1.39) vs. first tertile, *p* for trend 0.6
Wang et al., 2021, USA [61]	Prospective cohort; Nurses’ Health Study, two cohorts of Afro-American women from 14 states; 1431 ovarian cancer cases, 901 deaths from ovarian cancer; aged 25–42 years. Assessment every 2–4 years since 1986 in NHS I and 1989 in NHS II, with a median assessment of 4.6 years	Self-administered questionnaire on PA and sedentary behavior. Past-week recalls over 7 days. Recreational PA (average duration of eight common types of activity); estimated total weekly MET-hours.	Total PA (MET-h/wk) 1–8 years before diagnosis 1.5–<7.5 vs. <1.5 HR = 0.91 (0.68–1.22) ≥7.5 vs. <1.5, HR = 0.96 (0.72–1.27). Activity changes 1–8 years before diagnosis vs. 1–4 years after diagnosis: increased from <7.5 to ≥7.5, HR = 0.88 (0.58–1.35); decreased from ≥7.5 to <7.5, HR = 1.49 (1.07–2.08)
pancreatic			
Marshall et al., 2019, USA [48]	Retrospective cohort; Henry Ford Exercise Project; 49,143 adults, (46% women, 54% men), Lung cancer 282 deaths, colorectal cancer 89 deaths; mean age 54.0 years; 7.7-year follow-up	Bruce protocol treadmill exercise stress test (pick METs) based on achieved speed. Calculated by Quinton Controller and equations according to ACSM’s guidelines for exercise	Lung cancer: HR = 0.56 (0.32–1.00); colorectal cancer: HR = 0.11 (0.03–0.37) for the highest vs. the lowest tertile (≥12 vs. <6 MET). *p* for trends: 0.01 and <0.01, respectively
Cannioto et al., 2019, USA [62]	Prospective cohort; 5807 participants (55% women and 45% men) with 19 cancer types, from Roswell Park Comprehensive Cancer Center, 1956 deaths; mean age 60.63 years; 52.7-month follow-up	Self-administered Data Bank and BioRepository questionnaire; Questions on activity mode, frequency, intensity, and duration in the decade prior to study enrollment; MVPA assessed	HR for any regular/weekly MVPA: 0.68 (0.67–0.75) vs. no regular activity, HRs: 0.81 (0.69–0.95), 0.68 (0.60–0.0.78) and 0.85 (0.74–0.98) for engaging frequency: 1–2 days, 3–4 days, and 5–7 days, respectively
Stamatakis et al., 2022, UK [63]	Prospective cohort; UK Biobank Accelerometry Subsample; 22,699 nonexercising adults (56.2% women), 511 cancer death (13 cancer sites); mean age 61.8 years: 6.9-year follow-up	Daily vigorous intermittent lifestyle PA (VILPA), self-reported ≤ 1 min and ≤2 min duration bouts assessed using accelerometer	Three doses up to 1 min bout VILPA, HR = 0.60 (0.46–0.78), three doses up to 2 min, HR = 0.62 (0.48–0.80). VILPA duration: 4.4 min/day (up to 1 min bout): HR = 0.70 (0.59–0.84), 4.4 min/day (up to 2 bouts): HR = 0.70 (0.60–0.83)
Watts et al., 2022, USA [64]	Prospective cohort; National Institutes of Health—AARP Diet and Health Study Cohort: 272550 participants (58% men), 32,366 cancer deaths; mean age 70.5-year, 12.4-year follow-up	AARP Diet and Health Study questionnaire estimated weekly, duration and frequences of aerobics, exercise (e.g., running, cycling, swimming), racquet sports, golf, and walking for exercise. Activity estimated in MET-h/wk. Participation categorization in each activity type: nonpartcipant (control); 0.1–<7.5 (moderate active); 7.5–<15 (active); 15–<22.5 (highly active), ≥22.5 (very highly active)	HRs for total activity combination of the 7 activities: moderate active: 0.95 (0.94–0.97), active 0.87 (0.85–0.89), dose-response, compared with the first level. Racquet sports, running, aerobic exercise participation: HRs: 1.01 (0.85–1.21), 0.81 (0.69–0.95), and 0.91 (0.86–0.97), respectively
Chang et al., 2024, UK [65]	Prospective 490,659 participants from UK Biobank and 33,534 from NHANES datasets, 36,109 and 3057 deaths due to cancer, aged 37–73 years, 13.5- and 6.7-year follow-ups, respectively	Sedentary behavior determined by interview or self-assessment: time spent sitting or reclining per day (hour/day). PA assessment: walking for pleasure, light activity, strenuous sports and other activities (UK Biobank), recreational, household chores, yard work, walking, and bicycling daily duration	Subjects meeting the daily PA guidelines: sitting time 5–8 h/day vs. <5 h/day, HR = 1.034 (1.002–1.066) UK Biobank, HR = 1.072 (0.904–1.271) NHANES; >8 h/day, HR = 1.106 (1.047–1.167) UK Biobank, HR = 1.216 (0.977–1.513) NHANES. Replacing sedentary behavior with a 30 min/day PA, HR = 0.949 (0.943–0.955) UK Biobank, HR = 0.944 (0.933–0.957) NHANES, strenuous sports (60 min/day), HR = 0.923 (0.888–1.017) walking for pleasure (60 min/day), HR = 0.968 (0.936–1.000)
O’Donovan, 2024, Colombia [66]	Prospective Mexico City, 10023 subjects, mean age 53.3 years, 3409 deaths due to cancer, 17.6-year follow-up	Leisure-time PA (exercise and sports) frequency per week and duration using questionnaire. Categorization: no sport or exercise, “weekend” warrior (exercise and playing sports 1–2 times/wk), regularly active ≥ 3 times/wk	The weekend warrior group, HR = 0.82 (0.71–0.95), regularly active, HR = 0.94 (0.86–1.04) vs. non-sports exercise practice
Stamatakis et al., 2024, China [67]	Prospective longitudinal 349,248 participants aged ≥ 18 years, 4631 cancer deaths (men), and 3689 (women), 16.2- and 16.4-year follow-ups, respectively	Self-administered questionnaire, leisure-time PA in MET-h/wk: inactive (<1), low (1.00–7.49), moderate (7.5–14.99), high (≥15) (based on current PA guidelines). Occupational PA: light (mostly sedentary), moderately heavy/heavy (mostly standing or walking/loading or moving, heavy lifting)	Baseline occupational PA Moderate activity HR = 1.18 (0.97–1.43), moderately heavy/heavy HR = 1.11 (0.86–1.42) vs. light. Activity changes: decreased, HR = 1.20 (0.99–1.46); increased, HR = 1.07 (0.85–1.33) (in women)

**Table 4:** Summary of the most frequently proposed biological mechanisms for the role of physical activity in cancer disease prevention.

Potential Mechanisms	Effect of Physical Activity and Cancer Site
Reduced body fat and prevented weight gain	Activity reduces body fat, followed by decreasing levels of adipocytes, pro-inflammatory markers, estrogens, and exposure to bioavailable sex hormones (colon, postmenopausal breast, endometrium, ovaries).
Metabolic effects	Decreases C-peptide, insulin, IGF-1, fasting glucose levels, and fatty acids synthesis; increases glucose transport into muscle, muscle mass, IGFBP-3 level, insulin sensitivity; stimulates mitochondrial biogenesis, enhances cell resistance to environmental stressors (colon, postmenopausal breast, endometrium, prostate, ovaries, lung).
Hormonal effects	Regulates insulin resistance, reduces the level of bioavailable sex hormones, i.e., estrogens and androgens, decreases the level of free testosterone, and increases the formation of SHBG (breast, endometrium, ovaries, prostate).
Anti-inflammatory effects	Exercise decreases chronic inflammation by lowering levels of pro-inflammatory adipokines secreted by adipose tissue. Thus, it decreases levels of leptin and increases levels of adiponectin. Moreover, exercise decreases pro-inflammatory cytokines (IL-6, TNF-α, IL-1β), which can decrease CRP and serum amyloid levels (most cancers).
Innate immune system	Physical activity may trigger apoptosis, generate the responses of NK cells and lymphocytes, and thus enhance their activity in the immune system. It also participates in controlling cancerous and microbial cells, limiting their spread (most cancers).
Antioxidant refence, Prostaglandins	May control redox homeostasis by enhancing total endogenous antioxidant capacity, reduces oxidative stress by up-regulating levels of enzymatic antioxidants, e.g., CAT, GPX, SOD, and increases non-enzymatic antioxidants synthesis, e.g., glutathione, tocopherols, and stimulation of Vitamin D release from adipose tissue. Exercise may decrease DNA damage and enhance its repair. Exercise also inhibits the synthesis of prostaglandin E_2_ (promotor of colon cancer) and stimulates the synthesis of prostaglandin F2α, which is opposite to the effect of prostaglandin E_2_ (colon and most cancers).

## Data Availability

Data sharing is not applicable: Since this paper is a systematic review, no new data were generated. Thus, data sharing is not applicable to this article. Moreover, the data presented in this review were derived from the articles cited therein. Hence, the data are available in the respective journals indicated in the list of references.

## Abbreviations

ACSM	American College of Sports Medicine
BMI	Body Mass Index
CAT	catalase
CRP	C-reactive protein
GPAQ	Global Physical Activity Questionnaire
GTX	glutathione peroxidase
HR	Hazard Ratio
HRQoL	Health-related quality life
IGF-1	insulin-like growth factor-1
IGFBP-3	insulin growth factor-binding protein-3
IL-1β	interleukin-1β
IL-6	interleukin-6
IPAQ	International Physical Activity Questionnaire
LTPAQ	leisure-time physical activity questionnaire
MET	metabolic equivalent
MVPA	moderate-to-vigorous physical activity
OR	Odds Ratio
OS	oxidative stress
PA	physical activity
PAGAC	Physical Activity Guidelines Advisory Committee
PE	physical exercise
PI	physical inactivity
r	regression coefficient
RR	Relative Risk
SHBG	sex hormone-binding globulin
SOD	superoxidase
TNF-α	tumor necrosis factor-α
VILPA	vigorous intermittent lifestyle physical activity
WCRF/AICR	World Cancer Research Fund/American Institute for Cancer Research
